# Inverse association of marijuana use with nonalcoholic fatty liver disease among adults in the United States

**DOI:** 10.1371/journal.pone.0186702

**Published:** 2017-10-19

**Authors:** Donghee Kim, Won Kim, Min-Sun Kwak, Goh Eun Chung, Jeong Yoon Yim, Aijaz Ahmed

**Affiliations:** 1 Division of Gastroenterology and Hepatology, Stanford University School of Medicine, Stanford, California, United States of America; 2 Division of Gastroenterology and Hepatology, Department of Internal Medicine, Seoul National University College of Medicine, Seoul Metropolitan Government Boramae Medical Center, Seoul, Korea; 3 Department of Internal Medicine, Healthcare Research Institute, Gangnam Healthcare Center, Seoul National University Hospital, Seoul, Korea; Medizinische Fakultat der RWTH Aachen, GERMANY

## Abstract

**Background & aims:**

The impact of marijuana on nonalcoholic fatty liver disease (NAFLD) is largely unknown. We studied the association between marijuana and NAFLD utilizing cross-sectional data from the National Health and Nutrition Examination Survey (NHANES) from 2005–2014 and NHANES III (1988–1994).

**Methods:**

*Suspected* NAFLD was diagnosed if serum alanine aminotransferase (ALT) was > 30 IU/L for men and > 19 IU/L for women in the absence of other liver diseases (NHANES 2005–2014). In NHANES III cohort, NAFLD was defined based on ultrasonography.

**Results:**

Of the 14,080 (NHANES 2005–2014) and 8,286 (NHANES III) participants, prevalence of *suspected* NAFLD and ultrasonographically-diagnosed NAFLD were inversely associated with marijuana use (p < 0.001). Compared to marijuana-naïve participants, marijuana users were less likely to have *suspected* NAFLD (odds ratio [OR]: 0.90, 95% confidence interval [CI]: 0.82–0.99 for past user; OR: 0.68, 95% CI: 0.58–0.80 for current user) and ultrasonographically-diagnosed NAFLD (OR: 0.75, 95% CI: 0.57–0.98 for current user) in the age, gender, ethnicity-adjusted model. On multivariate analysis, the ORs for *suspected* NAFLD comparing current light or heavy users to non-users were 0.76 (95% CI 0.58–0.98) and 0.70 (95% CI 0.56–0.89), respectively (*P* for trend = 0.001) with similar trends in ultrasonographically-diagnosed NAFLD (OR: 0.77, 95% CI: 0.59–1.00 for current user; OR: 0.71, 95% CI: 0.51–0.97 for current light user). In insulin resistance-adjusted model, marijuana use remained an independent predictor of lower risk of *suspected* NAFLD.

**Conclusions:**

In this nationally representative sample, active marijuana use provided a protective effect against NAFLD independent of known metabolic risk factors. The pathophysiology is unclear and warrants further investigation.

## Introduction

In the past 20 years, the prevalence of nonalcoholic fatty liver disease (NAFLD) has increased dramatically to become the most prevalent liver disease in the United States (US).[1, 2] NAFLD represents a clinical spectrum ranging from nonalcoholic fatty liver to nonalcoholic steatohepatitis complicated by cirrhosis and hepatocellular carcinoma.[3] It is expected that increasing proportions of patients with NAFLD will develop cirrhosis and end-stage liver disease as they age. While lifestyle modifications such as weight loss and increasing physical activity are the cornerstone of medical management in patients with NAFLD, efforts are needed to identify and better understand other factors and measures that may prevent or retard the progression of NAFLD.

Marijuana is the most widely used illicit drug in the US, with an estimated 22.2 million current and past marijuana users based on the 2015 National Survey on Drug Use and Health.[4] About 1 in 5 young adults aged 18 to 25 (19.8%) reported current marijuana use, a rate considerably higher than those reported for adolescent (7.0%) and adults aged 26 or older (6.5%).[4] According to the National Epidemiologic Survey on Alcohol and Related Conditions, the prevalence of marijuana use more than doubled between 2001–2002 and 2012–2013.[5] The rising trend in marijuana use is expected to maintain its trajectory as a result of permissible regulatory environment. With the recent legalization of medical marijuana use across several US states, physicians increasingly encounter patients using marijuana and need to improve their medical awareness. Emerging data have suggested a paradox in which marijuana use is associated with increased appetite and calorie consumption, while the risk of obesity[6] and diabetes[7, 8] declined. Furthermore, data supporting the beneficial role of marijuana on insulin resistance are accumulating.[9, 10] Therefore, it can be hypothesized that marijuana use may have potential beneficial effects on metabolic abnormalities such as nonalcoholic fatty liver disease (NAFLD). Whether marijuana use plays a role in NAFLD pathogenesis via modification of *shared* risk factors, or by an independent pathway remains uncertain. In this population-based study, we assessed the association between marijuana use and NAFLD in the US.

## Methods

### Subjects and study design

This study represents analyses of the recent five 2-year cycles of the continuous National Health and Nutrition Examination Survey (NHANES) data between 2005 and 2014 and NHANES III data between 1988 and 1994. NHANES data from 2005–2006 to 2013–2014 were combined to increase the sample size and statistical reliability of the estimates. NHANES employs a stratified, multistage, clustered probability sampling design to reach a representative sample of the non-institutionalized civilian population in the US.

**Fig 1:**
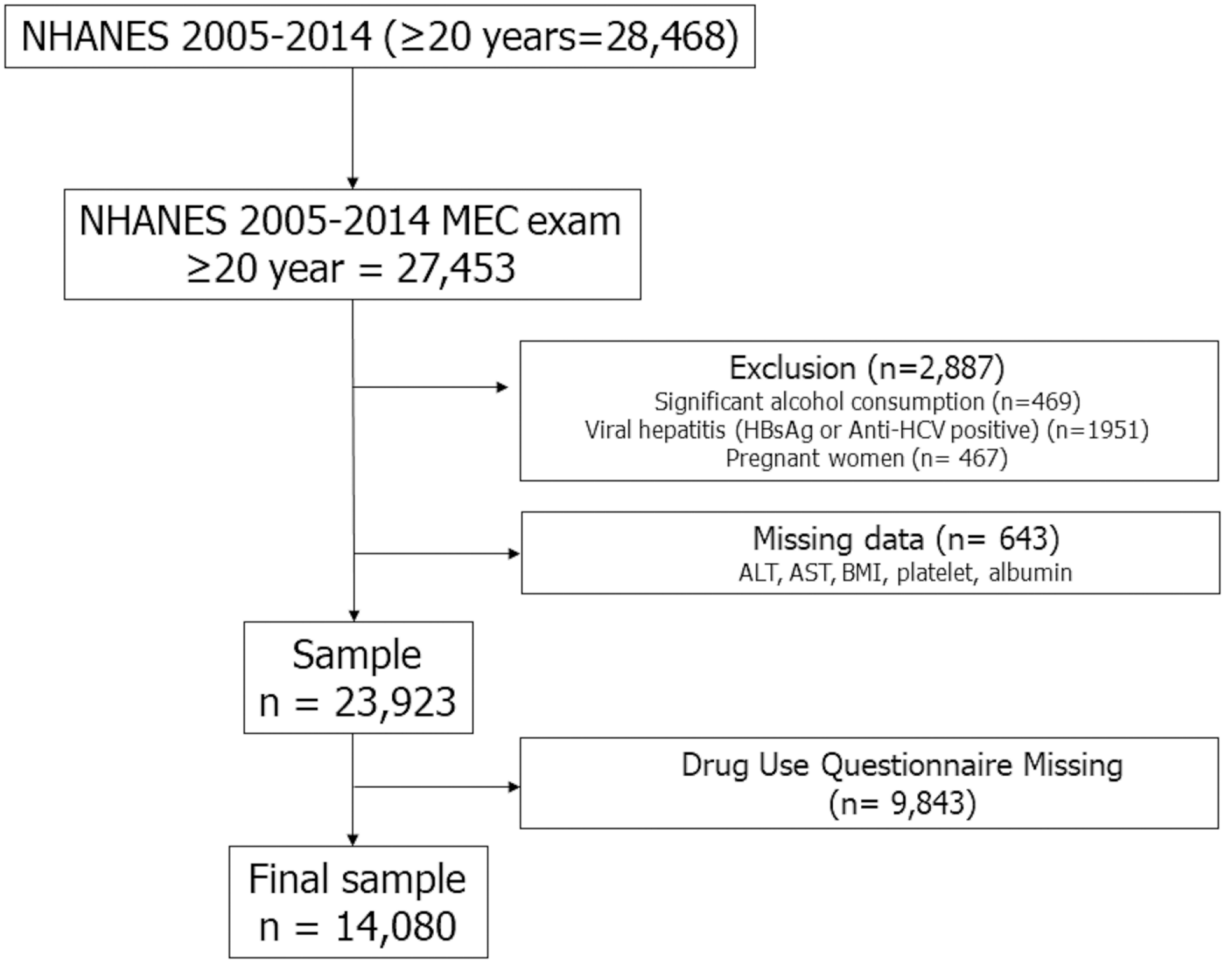
Flow diagram of participants for the study (NHANES 2005–2014).

**Fig 2:**
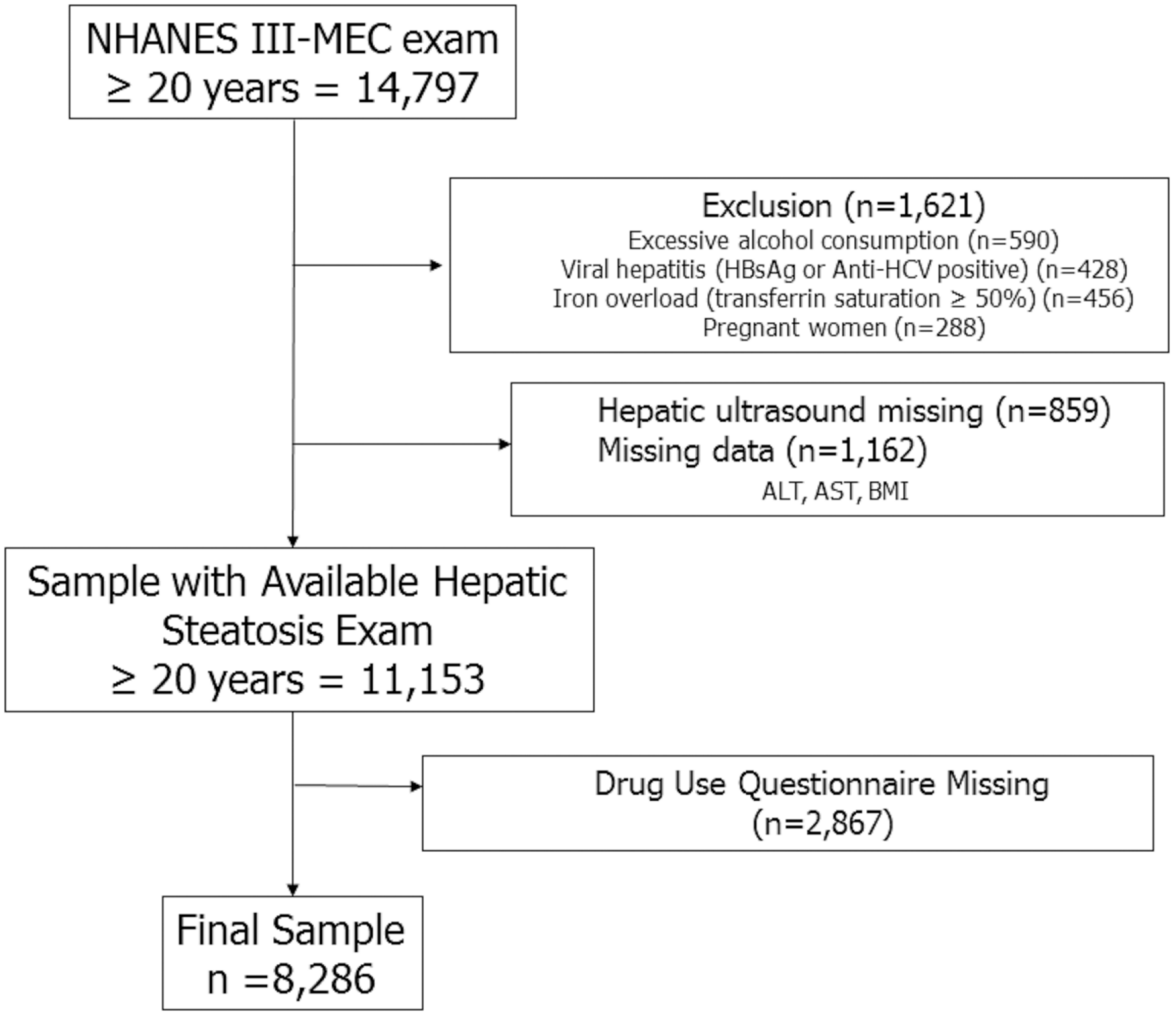
Flow diagram of participants for the study (NHANES III).

Of adult (≥ 20 years) participants in the NHANES 2005–2014 survey (n = 28,486), 96.4% (n = 27,453) underwent laboratory examination at a mobile examination center. Of those, 2,887 participants were excluded due to significant alcohol consumption (> 21 drinks/week in men and > 14 drinks/week in women), viral hepatitis (positive serum hepatitis B surface antigen and positive serum hepatitis C antibody), and pregnancy. In addition, we excluded 643 participants in whom data on serum aminotransferase, body mass index (BMI), and platelet count were not available. Of those, 9,843 participants were excluded in whom drug use questionnaire was not available. Thus, the final study sample consisted of 14,080 adults with complete data (Fig 1). Based on NHANES III database, among adult (≥ 20 years) participants (n = 14,797), we excluded subjects with significant alcohol consumption, viral hepatitis, iron overload (transferring saturation > 50%), and pregnancy (n = 1,621). We also excluded those with missing data on hepatic ultrasonography, serum aminotransferase, and body mass index (n = 2,021). Of those, 2,867 participants were excluded in whom drug use questionnaire was not available. Thus, the final study sample consisted of 8,286 adults (Fig 2).

The original survey was approved by the ethics review board of the National Center for Health Statistics and written informed consent for data collection was obtained from all participants. This analysis *per se* was deemed exempt by the Institutional Review Board at our institution, as the dataset used in the analysis was completely de-identified.

### Clinical and laboratory evaluations

A wide array of demographic information as well as anthropometric assessment and laboratory data were available in the final dataset. Race/ethnicity was categorized as non-Hispanic white, non-Hispanic black, Hispanic (Mexican-American, Other Hispanic), or others. Education was dichotomized with a cutoff set at high school graduation versus lack of high school graduation. Family income-to-poverty ratio categorized as ≤ 0.99 = below poverty; 1.00 and above = at or above poverty. Hypertension was diagnosed as systolic blood pressure ≥ 140 mmHg or diastolic blood pressure ≥ 90 mmHg and/or previous use of antihypertensive medication. Diabetes mellitus was defined as fasting plasma glucose concentration ≥ 126 mg/dl and/or treatment with a hypoglycemic agent or insulin. Iron overload was diagnosed at the transferrin saturation level of ≥ 50% (in NHANES III only). Smoking status was classified as never smokers, ex-smokers, and current smokers. Current smokers were defined as ongoing smoking or those who had smoked at least 100 cigarettes in the preceding 5 years. Alcohol consumption was calculated using self-reported data on the amount and frequency of alcohol use, as previously described.[11] Insulin resistance was evaluated using the homeostasis model assessment of insulin resistance (HOMA-IR), as described previously.[12]

### Definition of suspected NAFLD

*Suspected* NAFLD was defined to be serum alanine aminotransferase (ALT) > 30 U/L for men and > 19 for women [13, 14] in the absence of other known causes of chronic liver disease (significant alcohol consumptions, positive HBs Ag, positive anti-HCV, etc.). The laboratory analyses were performed to measure to serum ALT by using the Beckman Synchron LX20 and DxC800 UniCel systems (Beckman Coulter Inc, Brea, CA).

### Ultrasonographic examinations (NHANES III)

The technique used for hepatic steatosis in ultrasonography of the gall bladder images has been previously described.[15] The original NHANES III examination included ultrasonography of the gall bladder as a part of the assessment for digestive diseases in adults aged 20 to 74 years. Between 2009 and 2010, the archived gall bladder ultrasound video images were reviewed to assess fatty liver. Evaluation of the fatty liver was performed using the following five criteria: 1) parenchymal brightness, 2) liver to kidney contrast, 3) deep beam attenuation, 4) bright vessel walls, and 5) gallbladder wall definition. Assessment in these five categories was summarized to facilitate the diagnosis of hepatic steatosis. In addition to a dichotomous adjudication of presence/absence of steatosis, grading was provided as normal, mild, moderate, and severe. For the purpose of this study, NAFLD was defined as any degree (mild to severe) of steatosis.

### Marijuana use and other illicit drug use

In NHANES III, questionnaire data detailing the drug use were collected with paper questionnaire by interviewer. In contrast, Audio Computer-Assisted Self-Interview (ACASI) was used to obtain the drug use questionnaire data In NHANES 2005–2014. Marijuana use was defined from the following questions: (1) “Have you ever, even once, used marijuana or hashish?”, (2) “How long has it been since you last used marijuana or hashish?”, and (3) “During the past 30 days, on how many days did you use marijuana or hashish?”. Subjects were classified as never users (no lifetime use); past users (used previously but not within the last 30 days); current light user (at least once in the last 30 days but not on more than four different days); and current heavy user (at least five different days in the last 30 days). Current illicit drug use defined from the following questions: “During the past 30 days, on how many days did you use cocaine/ heroin/ amphetamine?” (current use of cocaine for NHANES III and NHANES 2005–2014; current use of heroin and amphetamine for NHANES 2005–2014 only).

### Statistical analysis

Given the complex sample design employed by NHANES, appropriate sample weights were used to reconstitute data on a population level for the entire US.[16] Frequencies of categorical variables and the means ± standard error of the continuous variables were calculated among comparison groups. Baseline characteristics were compared using the chi-square test for categorical variables or linear regression for continuous variables. Multivariable logistic models were created to identify predictors of NAFLD after consideration of other potential demographic and clinical confounders. All data analyses were implemented using STATA 13.0 (StataCorp, College Station, TX, USA) using Taylor series linearization.

## Results

Among the 14,080 participants (50% men; mean ages, 39.6 years) from NHANES 2005–2014, 38.0% (weighted proportion) had *suspected* NAFLD. The racial composition of the NHANES population was largely reflective of the US, including 67% non-Hispanic white, 11% non-Hispanic black, and 15% Hispanic participants. Table 1 tabulates characteristics of the NHANES population according to the marijuana use. Weighted proportions reporting never user or past user were 40.8% and 47.1%, whereas light current user and heavy current user were reported by 4.9% and 7.3% of American adults. There were noticeable differences in the demographic and clinical characteristics according to the marijuana use. Stratification by marijuana use status showed that young, male represented the majority of current marijuana users. Body mass index (BMI), waist circumference, prevalence of hypertension and diabetes were decreasing in tendencies with increasing marijuana use. Almost half of current marijuana users reported themselves as current smokers. Based on NHANES III database, among the 8,286 participants (48.3% men; mean ages, 37.6 years), 32.2% (weighted proportion) had *suspected* NAFLD. S1 Table showed characteristics of the NHANES population according to the marijuana use in this cohort. Weighted proportions reporting never user, past user and current user were 56.1%, 36.9%, and 7.0%, respectively. Within the 7% proportion of current marijuana users, light current user were 4.9% and heavy current user were 2.2% of American adults. Compared to NHANES 2005–2014, current marijuana heavy user sub-cohort was much smaller in NHANES III. Therefore, we decided to combine current heavy user and current light user into a consolidated current user category for further analysis. Other baseline characteristics did not differ significantly between NHANES 2005–2014 and NHANES III.

**Table 1:** Baseline characteristics of the study population according to marijuana use status (NHANES 2005–2014, n = 14,080).

Marijuana use	Never (n = 6,557)	Past user (n = 5,759)	Current user (n = 1,764)		
			Light user (n = 708)	Heavy user (n = 1,056)	
Age (years)	40.0 ± 0.3	40.6 ± 0.2	34.4 ± 0.6	34.0 ± 0.6	<0.001
Male (%)	43.8 ± 0.7	52.1 ± 0.7	56.5 ± 2.1	67.5 ± 1.7	<0.001
Body mass index (kg/m^2^)	29.2 ± 0.1	28.8 ± 0.1	27.3 ± 0.3	27.0 ± 0.2	<0.001
Waist circumference (cm)	96.8 ± 0.3	97.8 ± 0.3	93.1 ± 0.8	92.1 ± 0.7	<0.001
Hypertension (%)	21.1 ± 0.7	19.6 ± 0.6	14.8 ± 1.7	13.1 ± 1.3	<0.001
Diabetes (%)	7.9 ± 0.4	5.7 ± 0.4	3.7 ± 0.8	3.0 ± 0.6	<0.001
Ethnicity (%)					<0.001
Hispanics	23.3 ± 1.7	9.7 ± 0.8	12.8 ± 1.7	9.9 ± 1.1	
Non-Hispanic white	55.6 ± 2.2	76.7 ± 1.3	65.9 ± 2.3	68.8 ± 2.1	
Non-Hispanic black	11.2 ± 0.9	9.5 ± 0.7	16.7 ± 1.6	16.2 ± 1.5	
Asian/Other	10.0 ± 0.7	4.2 ± 0.3	4.6 ± 0.9	5.1 ± 0.8	
Smoking (%)					<0.001
Never	80.1 ± 0.8	45.2 ± 1.0	36.5 ± 2.7	23.0 ± 1.5	
Current smoker	10.0 ± 0.5	26.4 ± 1.0	46.0 ± 3.0	57.6 ± 2.3	
Ex-smoker	9.9 ± 0.6	28.4 ± 0.8	17.5 ± 1.8	19.5 ± 2.8	
High education (%)	82.1 ± 0.9	88.7 ± 0.8	84.5 ± 1.7	78.9 ± 1.8	<0.001
Married status (%)	68.5 ± 0.9	65.0 ± 1.0	44.9 ± 2.1	49.7 ± 2.1	<0.001
Poverty (%)	15.5 ± 0.9	11.4 ± 0.6	22.7 ± 1.9	23.6 ± 1.7	<0.001
Total cholesterol (mg/dL)	195.5 ± 0.8	197.3 ± 0.7	187.0 ± 1.9	190.0. ± 1.8	0.002
HDL-cholesterol (mg/dL)	51.6 ± 0.3	52.3 ± 0.3	53.3 ± 0.7	51.2 ± 0.5	0.318
HbA1c (%)	5.54 ± 0.01	5.43 ± 0.01	5.34 ± 0.04	5.37 ± 0.02	<0.001

Data are expressed as the mean ± SE or proportion ± SE. Abbreviation: NHANES, National Health and Nutrition Examination Survey; HDL cholesterol, high-density lipoprotein cholesterol. Marijuana use was shown as actual number.

As shown in Table 2, marijuana use categories were associated with a decreasing prevalence of *suspected* NAFLD and ultrasonographically-diagnosed NAFLD in a dose-dependent manner. In current heavy user category of marijuana, *suspected* NAFLD prevalence was 28.0%; while in current light user, past user, and never user categories the prevalence rates of *suspected* NAFLD were 30.5%, 38.0%, and 40.7%, respectively (p < 0.001). Consistently, prevalence of ultrasonographically-diagnosed NAFLD was 23.2% in current users of marijuana; while past users and never users demonstrated a NAFLD prevalence of 29.4% and 35.0%, respectively (p < 0.001). There were no significant age and gender-based differences.

**Table 2:** Prevalence of *suspected* NAFLD and ultrasonographically-diagnosed NAFLD stratified by marijuana use status.

	Never	Past user	Current user		
*Suspected* NAFLD using elevated ALT (NHANES 2005–2014)			Light user	Heavy user	P-value
NAFLD (%, 95% CI)	40.7 ± 0.8 (39.0–42.4)	38.0 ± 0.8 (36.5–39.6)	30.5 ± 2.2 (26.3–35.1)	28.0 ± 2.0 (24.2–32.1)	<0.001
Gender					
Men (%)	37.4	36.6	27.2	25.9	<0.001
Women (%)	43.3	39.6	34.8	32.4	0.002
Age					
-40 year (%)	37.8	35.1	27.4	25.7	<0.001
≥40 year (%)	43.4	40.3	36.5	32.9	0.001
Ultrasonography-diagnosed NAFLD (NHANES III)			Current user	P-value	
NAFLD (%, 95% CI)	35.0 ± 1.5 (31.9–38.1)	29.4 ± 2.1 (31.9–38.1)	23.2 ± 2.5 (18.2–28.2)	<0.001	
Gender					
Men (%)	38.8	30.2	21.6	<0.001	
Women (%)	32.2	28.4	24.5	0.039	
Age					
-40 year (%)	38.8	30.2	21.6	<0.001	
≥40 year (%)	32.2	28.4	24.5	0.039	

Data are expressed as the proportion ± SE. Abbreviation: NAFLD, nonalcoholic fatty liver disease; ALT, alanine aminotransferase; NHANES, National Health and Nutrition Examination Survey; CI, confidence interval

Results of logistic regression analyses with marijuana-naïve (without current or history of previous marijuana use) participants as the reference category can be seen in Table 3. Marijuana use categories were inversely associated with prevalence of NAFLD in the age, gender, and ethnicity-adjusted models. Current or past marijuana user was significantly associated with lower risk of the *suspected* NAFLD (odds ratio [OR]: 0.90, 95% confidence interval [CI]: 0.82–0.99 for past marijuana user, OR 0.68, 95% CI 0.58–0.80 for current marijuana user). After adjusting for BMI, educational level, economic status, smoking status, alcohol consumption, diabetes, hypertension, and current use of cocaine, heroin, and/or amphetamine, the ORs for *suspected* NAFLD comparing past marijuana user and current marijuana user to the never user categories were 0.90 (95% CI 0.81–1.00) and 0.71 (95% CI 0.58–0.85), respectively (*P* for trend = 0.001). The addition of total cholesterol and high-density lipoprotein cholesterol levels to the model did not significantly reduce the ORs for current marijuana user (*P* for trend = 0.001). With regard to the severity of current marijuana use, we analyzed multivariate models according to current light or heavy marijuana use (Table 3). In the age, gender, and ethnicity-adjusted analysis, current light and heavy use were inversely associated with *suspected* NAFLD (the ORs for *suspected* NAFLD comparing the current light and heavy marijuana users to marijuana-naive participants were 0.71 [95% CI 0.57–0.90] and 0.65 [95% CI 0.53–0.81], *P* for trend < 0.001). In the multivariate models, marijuana use categories were inversely associated with NAFLD in a dose-dependent fashion, with an adjusted OR comparing the light current user or heavy current user to never user of 0.72 or 0.70. After total cholesterol and HDL cholesterol were considered simultaneously in logistic regression model, the significance for heavy user persisted (OR 0.74, 95% CI 0.57–0.95, *P* for trend = 0.001). We then conducted a sensitivity analysis, in which a higher ALT cutoff defined as NHANES guideline (> 40 U/L for men and > 31 U/L for women) was used (S2 Table). The overall results remained similar–the inverse impact of marijuana on *suspected* NAFLD was weakened, partly because of a smaller number of participants in that category.

**Table 3:** Age, gender, ethnicity- adjusted and multivariate odds ratio of risk factor for the *suspected* NAFLD according to marijuana use (NHANES 2005–2014).

Marijuana use	Age, gender, ethnicity-adjusted		Multivariate model		Age, gender, ethnicity-adjusted		Multivariate model	
	OR (95% CI)	*P* value	OR (95% CI)	*P* value	OR (95% CI)	*P* value	OR (95% CI)	*P* value
Total population								
Never	1	<0.001*	1	0.001*				
Past user	0.90 (0.82–0.99)	0.025	0.91 (0.82–1.01)	0.079				
Current user	0.68 (0.58–0.80)	<0.001	0.73 (0.60–0.88)	0.001				
Total population								
Never	1	<0.001*	1	0.001*				
Past user	0.90 (0.82–0.99)	0.025	0.91 (0.82–1.01)	0.078				
Current user								
light user	0.71 (0.57–0.90)	0.004	0.76 (0.58–0.98)	0.038				
Heavy user	0.65 (0.53–0.81)	<0.001	0.70 (0.56–0.89)	0.003				
Men					Women			
Never	1	<0.001*	1	0.015*	1	0.012*	1	0.024*
Past user	0.98 (0.84–1.13)	0.756	0.98 (0.83–1.15)	0.811	0.85 (0.76–0.96)	0.008	0.87 (0.76–1.00)	0.052
Current user								
light user	0.62 (0.46–0.83)	0.002	0.75 (0.53–1.05)	0.093	0.85 (0.61–1.16)	0.300	0.81 (0.57–1.16)	0.247
Heavy user	0.58 (0.46–0.74)	<0.001	0.74 (0.57–0.95)	0.021	0.75 (0.53–1.05)	0.095	0.73 (0.50–1.05)	0.088
Age (<40 y)					Age (≥40 y)			
Never	1	<0.001*	1	0.001*	1	0.026*	1	0.092*
Past user	0.87 (0.77–0.99)	0.039	0.84 (0.72–0.97)	0.017	0.93 (0.81–1.05)	0.233	0.98 (0.84–1.13)	0.770
Current user								
light user	0.65 (0.49–0.86)	0.003	0.73 (0.55–0.98)	0.038	0.80 (0.51–1.26)	0.338	0.80 (0.48–1.29)	0.354
Heavy user	0.59 (0.45–0.76)	<0.001	0.65 (0.49–0.86)	0.003	0.71 (0.51–1.00)	0.050	0.74 (0.53–1.02)	0.067

Abbreviation: NAFLD, nonalcoholic fatty liver disease; NHANES, National Health and Nutrition Examination Survey; OR, odds ratio; CI, confidence interval. The multivariate model was adjusted for age, gender, ethnicity, education level, economic status, body mass index, smoking status, alcohol consumption, diabetes, hypertension, total cholesterol and high-density lipoprotein cholesterol, and current use of cocaine, heroin, and/or amphetamine.

*P value for the test of trend of odds.

Because gender-specific difference was important in the association between marijuana use and *suspected* NAFLD, subgroup analyses were conducted by gender. Among men, current heavy marijuana users demonstrated 26% reduction in the risk for prevalence of *suspected* NAFLD compared with non-users in the fully adjusted model (P for trend = 0.015). Among women, current heavy marijuana users showed a 27% reduction in the risk for *suspected* NAFLD with marginal significance. However, the association between marijuana use categories and *suspected* NAFLD remained significant even after full model adjustment (*P* for trend = 0.024). As there is a higher tendency of marijuana use in younger population, we conducted subgroup analyses by age (<40 vs. ≥40). Among younger population, current heavy marijuana users demonstrated 35% reduction in the risk for prevalence of *suspected* NAFLD compared with non-users in the fully adjusted model (P for trend = 0.001). Among older population, current heavy marijuana users showed a 26% reduction in the risk for *suspected* NAFLD with marginal significance (*P* = 0.067).

Current cigarette smoking was associated with decreased risk for *suspected* NAFLD (OR 0.82 95% CI 0.72–0.94) in the multivariate model. To determine interactive role between marijuana use and cigarette smoking for *suspected* NAFLD, the interaction between marijuana use and smoking on *suspected* NAFLD were assessed. As a result, there were no significant interactions between marijuana use and smoking for *suspected* NAFLD. When interaction term was added to the multivariate model, association between current marijuana use and NAFLD remained significant, whereas current cigarette smoking showed no significant association.

Results of logistic regression analyses using NHANES III database can be seen in Table 4. In the age, gender, and ethnicity-adjusted models, current marijuana users were significantly associated with lower risk of the ultrasonographically-diagnosed NAFLD (OR: 0.75, 95% CI: 0.57–0.98). After adjusting for BMI, educational level, economic status, smoking status, alcohol consumption, diabetes, hypertension, and current use of cocaine, this association persisted (OR: 0.77, 95% CI 0.59–0.99). The addition of total cholesterol levels to the model did not reduce the ORs for current marijuana user (OR: 0.77, 95% CI 0.59–1.00). In the multivariate models according to current light or heavy marijuana use, current light user was inversely associated with NAFLD (OR: 0.71, 95% CI: 0.51–0.97). Due to small number of current heavy user, there was no significant association in this model.

**Table 4:** Age, gender, ethnicity- adjusted and multivariate odds ratio of risk factor for the ultrasonography-diagnosed NAFLD according to marijuana use (NHANES III).

	Age, gender, ethnicity-adjusted		Multivariate model 1		Multivariate model 2	
	OR (95% CI)	*P* value	OR (95% CI)	*P* value	OR (95% CI)	*P* value
Marijuana use						
Never	1	0.039*	1	0.142*	1	0.151*
Past user	0.90 (0.74–1.11)	0.315	0.95 (0.77–1.18)	0.662	0.96 (0.77–1.18)	0.679
Current user	0.75 (0.57–0.98)	0.035	0.77 (0.59–0.99)	0.049	0.77 (0.59–1.00)	0.053
Marijuana use						
Never	1	0.075*	1	0.160*	1	0.170*
Past user	0.91 (0.75–1.12)	0.377	0.95 (0.77–1.18)	0.667	0.96 (0.77–1.19)	0.684
Current user						
light user	0.70 (0.52–0.95)	0.023	0.70 (0.51–0.97)	0.030	0.71 (0.51–0.97)	0.033
Heavy user	0.89 (0.52–1.53)	0.664	0.92 (0.54–1.57)	0.766	0.93 (0.55–1.57)	0.773

Abbreviation: NAFLD, nonalcoholic fatty liver disease; NHANES, National Health and Nutrition Examination Survey; OR, odds ratio; CI, confidence interval. The multivariate model 1 was adjusted for age, gender, ethnicity, education level, economic status, body mass index, smoking status, alcohol consumption, diabetes, hypertension, and current use of cocaine. The multivariate model 2 includes total cholesterol in addition to the variables addressed in model 1.

*P value for the test of trend of odds.

Among participants who fasted, a separate multivariable analysis that included serum triglycerides and insulin resistance as HOMA-IR showed these associations were attenuated but remained significant (*P* for trend = 0.012, Table 5). In the multivariate models, the OR for *suspected* NAFLD comparing participants who were current marijuana users to non-users was 0.72 (95% CI 0.55–0.94). When insulin resistance was considered in another logistic regression model, the significance for current light or heavy marijuana users persisted with marginal significance (0.71 [95% CI 0.49–1.02] for light users, 0.72 [95% CI 0.52–1.00] for heavy users, respectively, *P* for trend = 0.016). These results suggest that the association between current marijuana use (light versus heavy) and *suspected* NAFLD might be mediated, in part, by insulin resistance.

**Table 5:** Age, gender, ethnicity- adjusted and multivariate odds ratio of risk factor for the *suspected* NAFLD according to marijuana use in subjects with fasting data (NHANES 2005–2014, n = 6796).

	Age, gender, ethnicity-adjusted		Multivariate model 1		Multivariate model 2	
	OR (95% CI)	*P* value	OR (95% CI)	*P* value	OR (95% CI)	*P* value
Marijuana use						
Never	1	0.001*	1	0.012*	1	0.012*
Past user	0.83 (0.72–0.96)	0.011	0.84 (0.71–0.99)	0.040	0.87 (0.74–1.02)	0.089
Current user	0.71 (0.57–0.89)	0.004	0.73 (0.56–0.96)	0.025	0.72 (0.55–0.94)	0.015
Marijuana use						
Never	1	0.001*	1	0.018*	1	0.016*
Past user	0.83 (0.72–0.96)	0.011	0.84 (0.71–0.99)	0.040	0.87 (0.74–1.02)	0.090
Current user						
light user	0.74 (0.53–1.02)	0.065	0.74 (0.51–1.08)	0.121	0.71 (0.49–1.02)	0.066
Heavy user	0.69 (0.51–0.93)	0.015	0.73 (0.53–1.00)	0.052	0.72 (0.52–1.00)	0.049

Abbreviation: NAFLD, nonalcoholic fatty liver disease; NHANES, National Health and Nutrition Examination Survey; OR, odds ratio; CI, confidence interval. The multivariate model 1 was adjusted for age, gender, ethnicity, education level, economic status, body mass index, smoking status, alcohol consumption, diabetes, hypertension, total cholesterol, high-density lipoprotein cholesterol, fasting triglycerides, and current use of cocaine, heroin, and/or amphetamine. The multivariate model 2 includes HOMA index in addition to the variables addressed in model 1.

*P value for the test of trend of odds.

## Discussion

In this nationally representative survey of adults in the US, current marijuana use was associated with decreased risk of NAFLD independent of known metabolic risks. In our study, the lowest prevalence of NAFLD was noted in current heavy users of marijuana, while current light users and past users demonstrated intermediate risk of NAFLD with lower prevalence of NAFLD compared to non-users of marijuana. We found that past marijuana users had lower odds of *suspected* NAFLD than non-users, which suggests that previous exposure to marijuana may affect the development of NAFLD.

Against previous background of plausible harms from marijuana, metabolic risk estimates from current data suggest a protective effect. Recent studies report that marijuana use is associated with decreased risk of metabolic abnormalities such as obesity,[6] diabetes,[7, 8] and metabolic syndrome.[17] Based on the National Epidemiologic Survey on Alcohol and Related Conditions, current marijuana use (≥ 3 days/week) was associated with 39% reduction in the risk of obesity compared to no use during the past year.[6] In terms of diabetes risk, recent meta-analysis showed that the adjusted OR for diabetes was 0.7 (95% CI 0.6–0.8) for current marijuana users compared with never-users.[8] Even after adjusted for BMI, the association was maintained and remained significant.[8] Increasing amounts of data are now available supporting the inverse association between marijuana use and insulin resistance. Marijuana use was independently related to lower levels of fasting insulin and HOMA-IR compared to non-users from the nationally representative data.[9] Another study showed that adipocyte insulin resistance index was lower in current marijuana users compared with non-users.[10] The mechanism by which marijuana affects insulin resistance is not completely understood. Rimonobant, the cannabinoid type 1 receptor antagonist improved insulin sensitivity in experimental model.[18–20] One of the main active phytocannabinoid in marijuana, cannabidiol, can act as partial antagonist in cannabinoid type 1 receptor providing possible explanation for insulin sensitivity.[9, 20]

Current marijuana users had higher calorie and nutrients intake and more sodas and alcohol consumption than marijuana non-users.[21] Therefore, a heathier diet did not contribute to the lower prevalence of NAFLD among current marijuana users. In this study, current marijuana users seemed to have lower prevalence of obesity than marijuana non-users. Even though we adjusted for BMI which may be a potential protective factor for NAFLD, the inverse association between current marijuana users and prevalence of NAFLD persisted. However, when insulin resistance was considered in logistic regression model, the significance for current heavy or light marijuana user was attenuated but remained marginally significant. These results suggest that the association between current marijuana use and *suspected* NAFLD might be mediated, in part, by insulin resistance.

We may speculate and develop a hypothesis regarding the underlying pathophysiology supporting an inverse association between current marijuana use and NAFLD. Cannabinoids in marijuana have anti-inflammatory effects which favorably block inflammation through inhibitory effects on prostaglandin, histamine, and COX-2.[7, 22, 23] Cannabinoids antagonize release of many inflammatory cytokines and pro-inflammatory mediators, which may be implicated in pathological processes leading to insulin resistance and NAFLD, plausibly via cannabinoid-2 receptors.[24, 25] Experimental studies focusing on selective cannabinoid-2 receptor agonism have shown significant benefits by retarding the progression of diabetes [26, 27] and atherosclerosis [28] through the anti-inflammatory effects. Marijuana contains a variety of cannabinoids, of which some, such as cannabidiol and cannabigerol have antagonist properties that may mediate the anti-inflammatory properties of marijuana.[29] In addition, repeated administration of cannabinoids reduced cannabinoid-1 receptor density, which induced a tolerance.[30, 31] Thus, the downregulation of receptor may explain dose-dependent relationship between marijuana use and NAFLD in this study. Recent studies with a focus on inflammatory bowel disease have utilized non-psychotropic cannabinoids such as cannabidiol and cannabigerol.[23, 32] Therefore, non-psychotropic cannabinoid may be a potential candidate for the development of a new class of NAFLD drugs.

The strengths of our study are the utilization of high-quality data collected by trained personnel with a systematic protocol, wealth of metabolic variables, and a large number of subjects. Moreover, we believe that the subjects in our study are representatives of US general population. Therefore, the current findings are generalizable to the US population regardless of ethnicity/race. The use of ACASI program provided an ideal setting for sensitive information regarding marijuana use. Although we performed these analyses in a large representative sample of the general population, some limitations of our study merit comment. First, we used elevated serum ALT (in the absence of any other liver disease) to classify *suspected* NAFLD, which may underestimate the true prevalence of NAFLD in recent NHANES 2005–2014. We were able to further strengthen our conclusions when we reproduced the same results by analyzing a separate sub-cohort, NHANES III (1988–1994) using ultrasonographically-diagnosed NAFLD. Second, marijuana use based on self-report may be subject to misclassification and underestimation. However, ACASI interviews provide an environment for private interviews. It is possible that the lower prevalence of current heavy use of marijuana in NHANES III may have resulted from a difference in interviewing system between the two NHANES databases. Furthermore, while participants likely remember ever having used marijuana, their recollection of last use and number of days used in the previous 30 days may not be completely reliable. Such misreports could have introduced a bias toward the null hypothesis for the association between marijuana and NAFLD. However, it is likely that these misreports occurred comparably among all participants. Furthermore, NAFLD was defined as elevated ALT or ultrasonographic evidence of fatty liver after exclusion of significant alcohol consumption, viral hepatitis by serological testing, and iron overload. We were unable to exclude primary biliary cirrhosis, primary sclerosing cholangitis, Wilson’s disease, autoimmune liver disease, alpha-1-antitrypsin deficiency, and use of steatogenic medication due to limitation of NHANES database. In addition, a single measurement of serum ALT level could potentially lead to a misclassification as serum ALT levels can be intermittently or persistently normal in the setting of cirrhosis or chronic liver disease. However, we suspect that such change in serum ALT levels would bias our results toward null hypothesis and the true impact may be larger than what we reported. Finally, differential sampling error may affect comparisons over period because each cycle represents data from a different cross-sectional sample. However, these flaws are balanced by the benefits of a national, large population-based study and the ability to generalize these results to the population.

With these caveats in mind, we conclude that current marijuana use may favorably impact the pathogenesis of NAFLD in US adults. Current or active marijuana use was independently associated with lower risk of NAFLD, independent of metabolic risk factors. While the pathophysiology underlying this association remains to be elucidated, these data suggest cannabinoids to be explored as a possible strategy for the treatment and/or prevention of NAFLD. No recommendations can be made regarding the clinical application of marijuana in patients with NAFLD until prospective data are available.

## Supporting information

S1 Table Baseline characteristics of the study population according to marijuana use status (NHANES III, n = 8,286). (DOCX)

S2 Table Age, gender, ethnicity- adjusted and multivariate odds ratio of risk factor for the s*uspected* NAFLD (M>40, F>31) according to marijuana use. (DOCX)
