# Supplementary material for: Inverse association of marijuana use with nonalcoholic fatty liver disease among adults in the United States
Source: PLoS One. 2017 Oct 19;12(10):e0186702. doi: 10.1371/journal.pone.0186702 (PMC5648282; doi:10.1371/journal.pone.0186702)
Supplement: S1 Table — (DOCX) [file pone.0186702.s001.docx]

**S1 Table.** Baseline Characteristics of the Study Population According to Marijuana Use Status (NHANES III, n=8,286)

| Marijuana use | Never (n=5,227) | Past user (n=2,503) | Current user (n=556) | P-value |  |  | P-value^*^ |
| --- | --- | --- | --- | --- | --- | --- | --- |
|  |  |  |  |  | Light user (n=377) | Heavy user (n=179) |  |
| Age (years) | 40.8 ± 0.3 | 34.1 ± 0.3 | 30.5 ± 0.5 | <0.001 | 30.3 ± 0.6 | 30.9 ± 0.7 | <0.001 |
| Male (%) | 42.9 ± 0.8 | 53.9 ± 1.4 | 62.7 ± 3.4 | <0.001 | 60.3 ± 4.2 | 68.1 ± 5.6 | <0.001 |
| Body mass index (kg/m^2^) | 27.1 ± 0.2 | 26.0 ± 0.2 | 25.7 ± 0.4 | <0.001 | 25.3 ± 0.6 | 26.5 ± 0.5 | <0.001 |
| Waist circumference (cm) | 92.1 ± 0.4 | 89.4 ± 0.4 | 87.9 ± 1.1 | <0.001 | 86.7 ± 1.4 | 90.6 ± 1.6 | <0.001 |
| Hypertension (%) | 17.3 ± 0.9 | 9.9 ± 0.9 | 9.0 ± 2.0 | <0.001 | 10.4 ± 2.3 | 5.8 ± 2.5 | <0.001 |
| Diabetes (%) | 5.9 ± 0.5 | 2.3 ± 0.5 | 1.3 ± 0.6 | <0.001 | 1.2 ± 0.8 | 1.5 ± 0.8 | <0.001 |
| Ethnicity (%) |  |  |  |  |  |  | <0.001 |
| Hispanics | 11.4 ± 1.3 | 3.6 ± 0.6 | 7.1 ± 1.9 | <0.001 | 7.8 ± 2.3 | 5.5 ± 2.7 |  |
| Non-Hispanic white | 71.0 ± 1.7 | 82.1 ± 1.0 | 71.7 ± 2.6 |  | 71.8 ± 3.0 | 71.4 ± 3.8 |  |
| Non-Hispanic black | 10.3 ± 0.7 | 10.3 ± 0.8 | 17.5 ± 1.8 |  | 17.0 ± 2.2 | 18.4 ± 2.6 |  |
| Asian/Other | 7.4 ± 0.7 | 4.1 ± 0.3 | 3.8 ± 0.6 |  | 3.4 ± 0.7 | 4.7 ± 1.0 |  |
| Smoking (%) |  |  |  | <0.001 |  |  | <0.001 |
| Never | 58.7 ± 1.3 | 33.7 ± 2.0 | 26.8 ± 3.5 |  | 27.9 ± 3.9 | 24.2 ± 5.8 |  |
| Current smoker | 21.6 ± 1.2 | 37.8 ± 2.1 | 56.8 ± 3.4 |  | 54.1 ± 4.4 | 63.1 ± 5.0 |  |
| Ex-smoker | 19.8 ± 1.0 | 28.5 ± 1.3 | 16.4 ± 3.1 |  | 18.0 ± 3.9 | 12.8 ± 3.6 |  |
| High education (%) | 43.5 ± 1.6 | 52.3 ± 2.1 | 38.2 ± 3.6 | <0.001 | 37.8 ± 4.7 | 39.0 ± 5.6 | <0.001 |
| Married status (%) | 70.7 ± 0.9 | 68.9 ± 1.8 | 49.8 ± 3.2 | <0.001 | 46.0 ± 3.9 | 58.4 ± 5.7 | <0.001 |
| Poverty (%) | 12.3 ± 1.1 | 8.6 ± 0.7 | 17.4 ± 2.9 | <0.001 | 22.0 ± 4.0 | 7.3 ± 1.8 | <0.001 |
| Total cholesterol (mg/dL) | 203.4 ± 1.1 | 194.5 ± 1.3 | 188.2 ± 2.3 | <0.001 | 185.6 ± 3.0 | 193.8 ± 3.1 | 0.002 |
| HDL-cholesterol (mg/dL) | 49.6 ± 0.4 | 49.4 ± 0.6 | 51.7 ± 0.9 | 0.190 | 51.4 ± 1.2 | 52.6 ± 2.0 | 0.136 |
| HbA1c (%) | 5.36 ± 0.03 | 5.16 ± 0.03 | 5.17 ± 0.03 | <0.001 | 5.16 ± 0.03 | 5.19 ± 0.04 | <0.001 |
|  |  |  |  |  |  |  |  |

Data are expressed as the mean ± SE or proportion ± SE

Abbreviation: HDL cholesterol, high-density lipoprotein cholesterol

Marijuana use was shown as actual number.

^*^*P* value for comparison among 4 groups (Never vs. Past user vs. Light current user vs. Heavy current user).
