# Supplementary material for: Inverse association of marijuana use with nonalcoholic fatty liver disease among adults in the United States
Source: PLoS One. 2017 Oct 19;12(10):e0186702. doi: 10.1371/journal.pone.0186702 (PMC5648282; doi:10.1371/journal.pone.0186702)
Supplement: S2 Table — (DOCX) [file pone.0186702.s002.docx]

**S2 Table.** Age, Gender, Ethnicity- adjusted and Multivariate Odds Ratio of Risk Factor for the S*uspected* NAFLD (M>40, F>31) according to Marijuana Use

|  | Age, gender, ethnicity-adjusted |  | Multivariate model 1 |  | Multivariate model 2 |  |
| --- | --- | --- | --- | --- | --- | --- |
|  | OR (95% CI) | *P* value | OR (95% CI) | *P* value | OR (95% CI) | *P* value |
| Marijuana use |  | |  |  |  |  |
| Never | 1 | 0.008^*^ | 1 | 0.025^*^ | 1 | 0.062^*^ |
| Past user | 0.90 (0.81-1.01) | 0.068 | 0.87 (0.77-0.98) | 0.025 | 0.89 (0.79-1.00) | 0.045 |
| Current user | 0.76 (0.59-0.97) | 0.029 | 0.77 (0.58-1.02) | 0.072 | 0.82 (0.62-1.08) | 0.155 |
|  |  |  |  |  |  |  |
| Marijuana use |  | |  |  |  |  |
| Never | 1 | 0.008^*^ | 1 | 0.029^*^ | 1 | 0.062^*^ |
| Past user | 0.90 (0.81-1.01) | 0.068 | 0.87 (0.77-0.98) | 0.025 | 0.88 (0.79-1.00) | 0.044 |
| Current user |  |  |  |  |  |  |
| light user | 0.82 (0.55-1.22) | 0.331 | 0.81 (0.52-1.25) | 0.340 | 0.89 (0.57-1.39) | 0.599 |
| Heavy user | 0.72 (0.55-0.95) | 0.020 | 0.75 (0.55-1.01) | 0.057 | 0.77 (0.58-1.04) | 0.088 |
|  |  |  |  |  |  |  |

Abbreviation: NAFLD, nonalcoholic fatty liver disease; OR, odds ratio; CI, confidence interval

The multivariate model 1 was adjusted for age, gender, ethnicity, education level, economic status, body mass index, smoking status, alcohol consumption, diabetes, hypertension, and current use of cocaine, heroin, and/or amphetamine.

The multivariate model 2 includes total cholesterol and high-density lipoprotein cholesterol in addition to the variables addressed in model 1.
